# Diagnostic performance of standardized typical CT findings for COVID-19: a systematic review and meta-analysis

**DOI:** 10.1186/s13244-023-01429-2

**Published:** 2023-05-24

**Authors:** Bo Da Nam, Hyunsook Hong, Soon Ho Yoon

**Affiliations:** 1 Department of Radiology, Soonchunhyang University College of Medicine, Soonchunhyang University Seoul Hospital, Seoul, Republic of Korea; 2 Medical Research Collaborating Center, Seoul National University Hospital, Seoul, Republic of Korea; 3 Department of Radiology, Seoul National University College of Medicine, Seoul National University Hospital, Seoul, Republic of Korea

**Keywords:** COVID-19, Pneumonia (viral), Tomography (X-Ray Computed), Lung, Meta-analysis

## Abstract

**Objective:**

To meta-analyze diagnostic performance measures of standardized typical CT findings for COVID-19 and examine these measures by region and national income.

**Methods:**

MEDLINE and Embase were searched from January 2020 to April 2022 for diagnostic studies using the Radiological Society of North America (RSNA) classification or the COVID-19 Reporting and Data System (CO-RADS) for COVID-19. Patient and study characteristics were extracted. We pooled the diagnostic performance of typical CT findings in the RSNA and CO-RADS systems and interobserver agreement. Meta-regression was performed to examine the effect of potential explanatory factors on the diagnostic performance of the typical CT findings.

**Results:**

We included 42 diagnostic performance studies with 6777 PCR-positive and 9955 PCR-negative patients from 18 developing and 24 developed countries covering the Americas, Europe, Asia, and Africa. The pooled sensitivity was 70% (95% confidence interval [CI]: 65%, 74%; *I*^2^ = 92%), and the pooled specificity was 90% (95% CI 86%, 93%; *I*^2^ = 94%) for the typical CT findings of COVID-19. The sensitivity and specificity of the typical CT findings did not differ significantly by national income and the region of the study (*p* > 0.1, respectively). The pooled interobserver agreement from 19 studies was 0.72 (95% CI 0.63, 0.81; *I*^2^ = 99%) for the typical CT findings and 0.67 (95% CI 0.61, 0.74; *I*^2^ = 99%) for the overall CT classifications.

**Conclusion:**

The standardized typical CT findings for COVID-19 provided moderate sensitivity and high specificity globally, regardless of region and national income, and were highly reproducible between radiologists.

**Critical relevance statement:**

Standardized typical CT findings for COVID-19 provided a reproducible high diagnostic accuracy globally.

**Key points:**

- Standardized typical CT findings for COVID-19 provide high sensitivity and specificity.
- Typical CT findings show high diagnosability regardless of region or income.
- The interobserver agreement for typical findings of COVID-19 is substantial.

**Graphical abstract:**

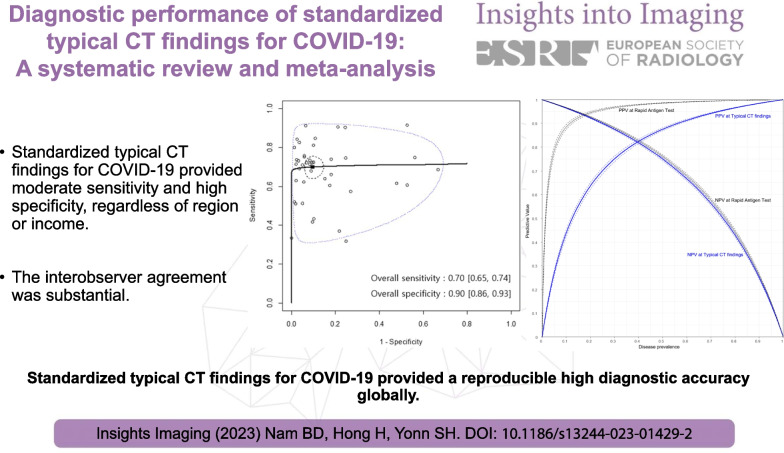

**Supplementary Information:**

The online version contains supplementary material available at 10.1186/s13244-023-01429-2.

## Introduction

The ongoing COVID-19 pandemic, caused by the novel virus SARS-CoV-2, has affected all countries worldwide [1]. As of September 26, 2022, the World Health Organization had reported more than 600 million confirmed cases of COVID-19 and 6.5 million COVID-19-related deaths (World Health Organization dashboard, accessed September 26, 2022). During the pandemic, early and accurate diagnoses of COVID-19 have been crucial for preventing the spread of infection and managing patients promptly.

The reference standard for COVID-19 has been the reverse-transcription polymerase chain reaction (RT-PCR) test using respiratory tract specimens. Despite the high diagnostic accuracy of RT-PCR, it was reported that 20–67% of infected patients had false-negative results, and turnaround times ranged from 6 h to over 48 h in the early pandemic [2]. Moreover, RT-PCR could not differentiate between infectious and non-infectious SARS-CoV-2 particles [3, 4]. The rapid antigen test (RAT) provided results faster than RT-PCR and enabled rapid point-of-care triage [5, 6], but RAT was not available in the early stages of the pandemic.

Chest CT imaging has been widely used for COVID-19 since the initial stages of the pandemic of COVID-19. A meta-analysis of early diagnostic CT studies in April 2020 showed that unstandardized CT interpretation was sensitive but nonspecific to COVID-19 [7]. Subsequently, two major CT classification systems for COVID-19 were proposed for standardized interpretation according to the typicality of CT findings: the four-category Radiological Society of North America (RSNA) classification system [8] and the five-category COVID-19 Reporting and Data System (CO-RADS) [9]. The RSNA and CO-RADS classification systems exhibit a high degree of similarity. Specifically, CO-RADS categories 1, 2, 3–4, and 5 correspond, respectively, to the negative, atypical, indeterminate, and typical categories of the RSNA classification system. Early analysis including nine studies, mostly from developed European countries, showed the possibility that each standardized system might better diagnostic performance than unstandardized CT interpretation [10]. Nevertheless, radiology human and facility resources for COVID-19 differed across countries [11], calling into question whether this standardized interpretation of typicality worked similarly worldwide.

This study aimed to meta-analyze diagnostic performance measures of the standardized typical CT findings for COVID-19 and examine these measures by region and national income.

## Materials and methods

We conducted this systematic review and meta-analysis according to the Preferred Reporting Items for Systematic Reviews and Meta-Analyses guidelines [12].

### Search strategy

A search of the OVID-MEDLINE and Embase databases was conducted for publications on the diagnostic performance of the RSNA classification and the CO-RADS systems in patients with COVID-19 infection. The following keywords were used in different combinations: (“Radiological Society of North America” OR “RSNA” OR “CO-RADS” OR “CORADS”) AND (“Corona” OR “Coronavirus” OR “COVID-19” OR “SARS-Cov-2” OR “2019nCoV”). The search was restricted to human subjects and English-language studies. In addition, publications that cited the original RSNA classification system [8] and CO-RADS [9] for reporting COVID-19 pneumonia were also searched using the “cited reference” function in OVID- MEDLINE and Embase. The search was updated until April 6, 2022.

### Study selection

Original studies were eligible for inclusion if they provided data on the diagnostic performance of the RSNA classification or CO-RADS system in evaluating patients with clinically suspected COVID-19 infection, using RT-PCR as the reference standard. Among them, we included only studies from which diagnostic performance measures (sensitivity and specificity) or the interobserver agreement of the CT classification (relative to RT-PCR) could be extracted. The exclusion criteria were: (1) case reports, review articles, editorials, letters, comments, and conference proceedings; (2) studies with insufficient data to compose a 2-by-2 contingency table to calculate sensitivity and specificity on per-patient level for either the RSNA classification or CO-RADS system; and (3) studies that only provided data on the performance of an artificial intelligence-based analysis. The full texts of the articles were reviewed after selecting potentially eligible abstracts.

### Study data extraction

Two of the authors (B.D.N. and S.H.Y.) with 11 and 18 years of clinical experience, respectively, independently extracted the data using a standardized form: (1) patient characteristics; (2) study characteristics; (3) the results of each diagnostic test regarding the results of RT-PCR assays; and (4) the interobserver agreement for the RSNA classification and the CO-RADS system. The two systems identically defined typical CT findings for COVID-19 with different labels as a typical appearance in the RSNA system and grade 5 in the CO-RADS system [9]. Accordingly, we regarded the typical appearance in the RSNA and grade 5 in the CO-RADS system as standardized typical CT findings for COVID-19.

National income was subcategorized into low-income, lower-middle-income, upper-middle-income, and high-income economies based on World Bank data [13]. Developing countries were defined as low- to upper-middle-income economies, and developed countries were defined as high-income economies. Country-level rates of COVID-19 vaccination were sourced from the Our World in Data [14]. Full vaccination was defined as the completion of two or more doses.

### Definition of outcomes

The primary outcome of this meta-analysis was the pooled diagnostic performance of typical CT findings (“typical” in the RSNA CT classification or CO-RADS 5). The RSNA and CO-RADS CT classification systems are similar and our study analyzed that CO-RADS categories 1, 2, 3–4, and 5 were interpreted respectively as negative, atypical, indeterminate, and typical categories of the RSNA system, in accordance with previous research [10]. To assist with interpretation, representative CT images were included in the Appendix (Additional file 1: Fig. S1). Secondary outcomes comprised the interobserver agreement for standardized CT classification systems.

**Fig. 1:**
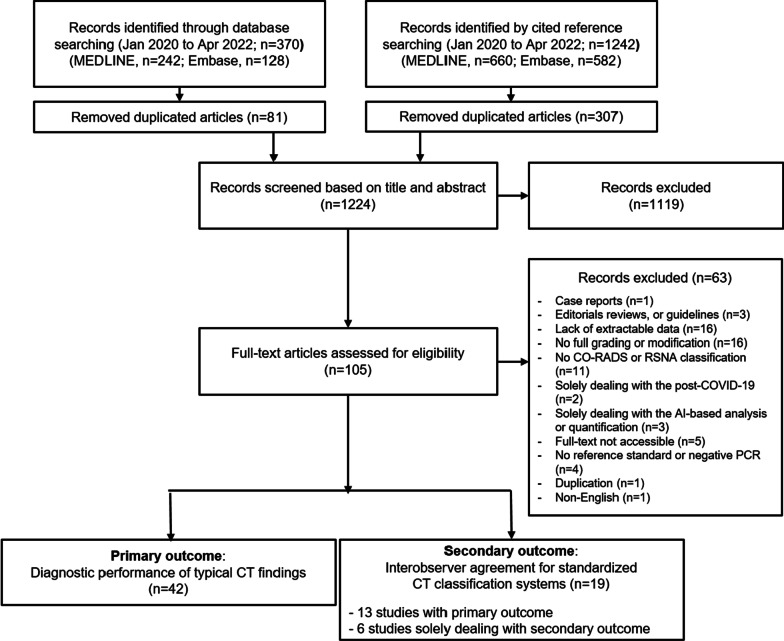
Flow diagram of study selection

### Study quality assessment

The quality of included studies was assessed by two of the authors (B.D.N. and S.H.Y.) using the Quality Assessment of Diagnostic Accuracy Studies 2 (QUADAS-2) tool, which comprises four key items: patient selection, index test, reference standard, and flow and timing [15].

### Statistical analysis

The meta-analysis of diagnostic accuracy was performed using a bivariate generalized linear mixed model, which models sensitivity and specificity jointly and yields unbiased estimates with sparse data [16]. Summary values for sensitivity and specificity, a 95% confidence region for the summary values, and a 95% prediction region were estimated, and the summary receiver-operating characteristic (ROC) curve was derived from the bivariate model. The *I*^2^ statistic was used to assess heterogeneity across the studies. The source of between-study variability was explored by including study-level characteristics in the model, and the characteristics were CT classification system, prevalence, mean age, sex (proportion of male subjects), study size, national income, regions (continent), and full vaccination rate. A sensitivity analysis was conducted for studies with more than 50 subjects in both COVID-19 cases and non-COVID-19 patients. The positive predictive value (PPV) and negative predictive value (NPV) of the typical CT findings were estimated and compared to those of RAT [17]: the pooled sensitivity of was 69% (95% confidence interval [CI]: 68%, 70%; *I*^2^ = 96%), and the pooled specificity was 99% (95% CI 99%, 99%; *I*^2^ = 94%).

In addition, a meta-analysis of observer agreement was conducted for binary classification between typical versus non-typical findings and overall classification. The risk of publication bias in studies reporting diagnostic accuracies and interrater agreement was assessed using Deeks’ regression test. The analysis was done using the *lme*, *altmeta*, and *metafor* packages in R (version 4.1.2).

## Results

### Literature search

Our literature search process is outlined in Fig. 1. In total, 1,224 articles were screened after the removal of duplicate articles. Of these 1224 articles, 1119 were excluded based on their titles and abstracts. Sixty-three additional articles were excluded after reviewing their full texts, resulting in a total of 46 articles that were finally included: 42 studies for assessing diagnostic accuracy and 19 studies for assessing the interobserver agreement [9, 18–64].

### Baseline characteristics

The characteristics of the included studies are outlined in Table 1. Among 42 studies for assessing diagnostic accuracy, the study population inclusion period ranged from January 2020 to August 2021. The total number of the included patients was 16,732 from 16 countries, of whom 6777 had positive RT-PCR and 9,955 had negative RT-PCR results (median patient number, 229; interquartile range, 109 to 526 patients). The median and interquartile range of COVID-19 prevalence were 45.5% and 31.9% to 61.2%, respectively. Thirty-nine studies were conducted when vaccination was not available. There were five prospective studies, and most were retrospective studies. The mean or median age of patients ranged from 40 to 76 years old. There were 18 studies from developing countries (4 studies from lower-middle-income and 14 studies from upper-middle-income countries) [18, 19, 22, 23, 30–32, 35, 37, 38, 43–45, 47, 48, 50, 51, 58] and 24 studies from developed countries (high income) [9, 20, 21, 24–29, 33, 34, 36, 39–42, 46, 49, 52–57]. The included studies were predominantly from Europe (24 studies) [9, 20, 21, 23–27, 29, 31, 32, 34, 35, 37, 38, 41, 42, 47–49, 52, 55–57], followed by the Americas (10 studies), Asia (seven studies), and Africa (one study). Nineteen studies for assessing the interobserver agreement are summarized in Additional file 1: Table 1.

**Table 1:** Study characteristics reporting diagnostic accuracy of typical CT findings

First author	Country	Continent	National income	Study design	Patient number	Inclusion period	Mean Age (y)	Male (%)	COVID-19 prevalence (%)	CT sensitivity	CT specificity
Abdolrahimzadeh	Iran	Asia	Low-middle	Retrospective	86	Feb to May, 2020	41 ± 20	83	26	0.32	0.75
Barbosa	Brazil	America	Upper-middle	Retrospective	91	Feb to Mar, 2020	58	60	28	0.64	0.85
Bellini	Italy	Europe	High	Retrospective	572	Mar to May, 2020	63 ± 20	58	25	0.41	0.90
Berkel	Belgium	Europe	High	Retrospective	200	Mar to Apr, 2020	67 ± 17	48	34	0.74	0.92
Borges	Brazil	America	Upper-middle	Retrospective	175	Mar, 2020	43 ± 21	59	50	0.74	0.98
Cengel	Turkey	Europe	Upper-middle	Retrospective	534	Apr, 2020	48 ± 17	57	74	0.60	0.82
Ciccarese	Italy	Europe	High	Retrospective	460	Feb to Mar, 2020	64	59	46	0.72	0.92
De Jaegere	Netherlands	Europe	High	Retrospective	96	Mar, 2020	70	64	47	0.51	0.98
De Smet	Belgium	Europe	High	Prospective	859	Mar to Apr, 2020	70	52	42	0.69	0.96
Fujioka	Japan	Asia	High	Retrospective	154	Apr to June, 2020	61 ± 19	66	49	0.52	0.95
Gross	Germany	Europe	High	Retrospective	96	Mar to Apr, 2020	64	52	21	0.80	0.99
Guimaraes	Brazil	America	Upper-middle	Prospective, Retrospective	492	Mar to Dec, 2020	65	55	89	0.87	0.93
Gümüs	Turkey	Europe	Upper-middle	Retrospective	218	Apr to May, 2020	56 ± 16	52	1	0.33	1.00
Hammer	USA	America	High	Retrospective	2618	Apr to Mar, 2021	65	55	13	0.68	0.91
Hermans	Netherlands	Europe	High	Prospective	319	Mar to Apr, 2020	59	56	42	0.75	0.94
Inanc	Turkey	Europe	Upper-middle	Retrospective	665	May to Oct, 2020	55	54	34	0.37	0.78
Inui	Japan	Asia	High	Retrospective	100	Jan to June. 2020	59 ± 17	76	50	0.74	0.83
Kavak	Turkey	Europe	Upper-middle	Retrospective	903	July, 2020	49 ± 17	49	68	0.81	0.90
Koşar	Turkey	Europe	Upper-middle	Retrospective	209	Mar to May, 2020	57	55	71	0.72	0.90
Kuokawa	Japan	Asia	High	Retrospective	154	Apr to May, 2020	65	70	17	0.83	0.89
Lieveld	Netherlands	Europe	High	Prospective	741	Mar to May, 2020	62.1 ± 17	56	32	0.72	0.93
Majeed	UK	Europe	High	Prospective	259	Mar to May, 2020	NS	NS	15	0.58	0.73
Miranda	Brazil	America	Upper-middle	Retrospective	71	Mar, 2020	47	47	45	0.83	0.97
Nair	India	Asia	Low-middle	Retrospective	164	Apr to July, 2020	49 ± 15	84	62	0.73	0.96
O’ Neill	Canada	America	High	Retrospective	279	Feb to May, 2020	60 ± 17	51	70	0.72	0.90
Özel	Turkey	Europe	Upper-middle	Retrospective	280	Mar to Apr, 2020	46 ± 16	54	40	0.75	0.44
Özer	Turkey	Europe	Upper-middle	Retrospective	1186	Mar, 2020	47 ± 19	55	32	0.63	0.98
Palmisano	Italy	Europe	High	Retrospective	142	Mar, 2020	61	71	55	0.90	0.76
Palwa	Pakistan	Asia	Low-middle	Retrospective	500	Apr to Jun, 2020	55 ± 14	81	85	0.62	0.52
Prokop	Netherlands	Europe	High	Retrospective	105	Mar, 2020	62 ± 16	58	51	0.62	0.95
Rocha	Brazil	America	Upper-middle	Retrospective	160	May to Jun, 2020	59	53	49	0.52	0.98
Schalekamp	Netherlands	Europe	High	Retrospective	1070	Mar to Apr, 2020	66	59	50	0.71	0.95
Shirota	Japan	Asia	High	Retrospective	66	May to Jun, 2020	76	65	32	0.71	0.98
Silva	Chile	America	High	Retrospective	240	Mar to Apr, 2020	48 ± 156	37	36	0.76	0.94
Turcato	Italy	Europe	High	Retrospective	120	Mar, 2020	68	61	43	0.75	0.75
Valentin	Germany	Europe	High	Retrospective	536	Mar to Nov, 2020	69 ± 16	63	21	0.83	0.95
Vicini	Italy	Europe	High	Retrospective	714	Mar to Jul, 2020	64 ± 19	59	37	0.44	0.90
Yassin	Egypt	Africa	Low-middle	Retrospective	144	Nov, 2020 to Feb, 2021	58	59	69	0.66	0.82
Falaschi	Italy	Europe	H igh	Retrospective	773	Mar to Apr, 2020	62 ± 18	55	60	0.91	0.79
Guner	Turkey	Europe	Upper-middle	Retrospective	104	June to Aug, 2021	64 ± 17	59	64	0.61	0.47
Lang	USA	America	High	Retrospective	22	Mar, 2020	64 ± 16	55	73	0.69	0.33
Martinez-Fierro	Mexico	America	Upper-middle	Retrospective	55	Nov, 2020 to May, 2021	57 ± 17	56	66	0.92	0.47

NS, not specified

### Quality assessment

The included studies for reporting diagnostic accuracy and interobserver agreement had a relatively low risk of bias in flow and timing, reference standard, and patient selection (Additional file 1: Fig. 2). In regard to the index test, five of 48 studies [18, 19, 25, 59, 63] did not perform RT-PCR result blinding during the interpretation of CT images, causing a high risk of bias. Fifteen of 48 studies [29, 31–34, 42, 43, 46, 47, 49, 50, 55, 56, 58, 60] lacked a description of blinding regarding the RT-PCR results, for which reason the risk of bias was deemed unclear in those studies.

**Fig. 2:**
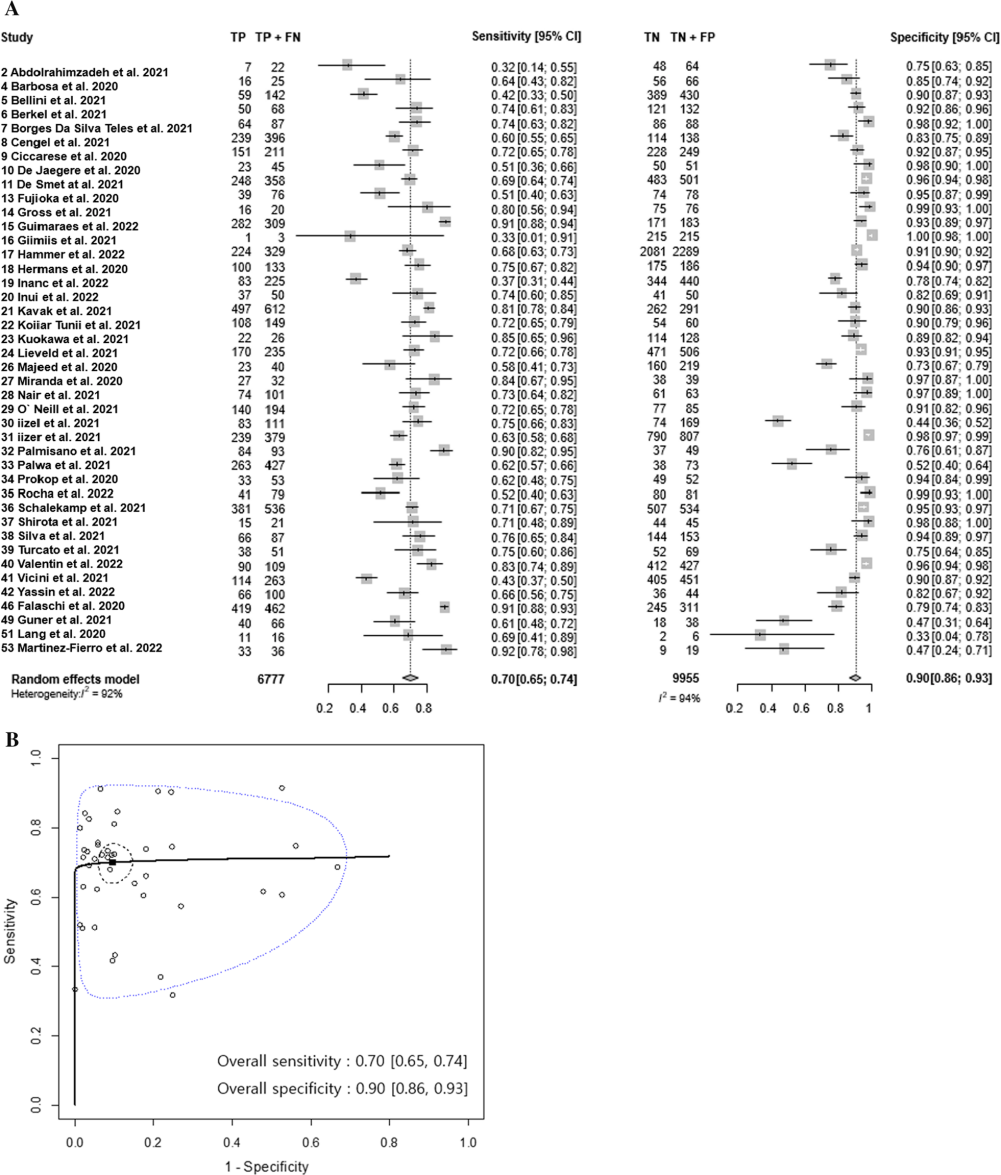
**A** Coupled forest plot for the diagnostic accuracy of typical CT findings. **B** Summary ROC curve in studies reporting both sensitivity and specificity

### Diagnostic performance of typical CT findings of COVID-19

The pooled sensitivity was 70% (95% CI 65%, 74%; *I*^2^ = 92%) and the pooled specificity was 90% (95% CI 86%, 93%; *I*^2^ = 94%) for the typical CT findings of COVID-19 (Fig. 2). There was high heterogeneity for both sensitivity and specificity (proportion of prediction region to the area under the ROC curve = 35.8%). The pooled prevalence of COVID-19 was 44.5% (95% CI 37.4%, 51.8%; *I*^2^ = 99%).

Meta-regression analyses for the sensitivity and specificity of the typical CT findings (Table 2) revealed that as the prevalence rate increased, the sensitivity tended to increase (*p* 0.05), and the specificity significantly decreased (*p* 0.01) (Additional file 1: Fig. 3). The sensitivity of the typical CT findings for COVID-19 did not differ by national income (developing countries, 68% (95% CI 61%, 75%); developed countries, 71% (95% CI 65%, 77%; *p* 0.57) and the region of the study (*p* 0.25). There was no significant difference in the specificity of the typical CT findings for COVID-19 by national income (developing countries, 89% (95% CI 81%, 93%); developed countries, 91% (95% CI 87%, 95%; *p* 0.43) and the region of the study (*p* 0.85). The diagnostic specificity of the typical CT findings for COVID-19 in studies of vaccinated patients was significantly lower than in studies of unvaccinated patients (68% vs. 91%, *p* 0.03). There was no significant difference in diagnostic performance according to the mean age, the proportion of male patients, study size, or CT classification system (*p* > 0.1, respectively). A sensitivity analysis confined to large studies showed similar pooled sensitivity and specificity (Additional file 1).

**Table 2:** Pooled estimates and meta-regression of diagnostic accuracy of typical CT findings

	Number of studies	Sensitivity [95% CI]	*p* value†	Specificity [95% CI]	*p* value†
Overall	42	0.70 [0.65, 0.74]		0.90 [0.86, 0.93]	
*Meta-regression*					
Prevalence (%)^‡^		0.01 [0.00, 0.02]	0.0494	− 0.02 [− 0.04, − 0.01]	0.0121
Mean age (years)^‡^		0.01 [− 0.02, 0.04]	0.4880	0.02 [− 0.03, 0.07]	0.4850
Proportion of male subjects (%)^‡^		− 0.01 [− 0.04, 0.01]	0.3372	− 0.03 [− 0.07, 0.01]	0.1435
Study size^‡^		0.00 [− 0.03, 0.02]	0.7879	0.02 [− 0.02, 0.06]	0.4266
National income			0.4453		0.4219
Lower-middle $1086–4255	4	0.60 [0.43, 0.75]		0.81 [0.56, 0.94]	
Upper-middle $4256–13,205	14	0.71 [0.62, 0.78]		0.90 [0.83, 0.95]	
High > $13,205	24	0.71 [0.65, 0.77]		0.91 [0.85, 0.95]	
National income			0.5707		0.4349
Developing	18	0.68 [0.61, 0.75]		0.89 [0.81, 0.93]	
Developed	24	0.71 [0.65, 0.77]		0.91 [0.87, 0.95]	
Region of the study			0.2532		0.8451
Asia & Africa	8	0.65 [0.53, 0.76]		0.88 [0.75, 0.95]	
Europe	24	0.69 [0.63, 0.74]		0.91 [0.86, 0.94]	
America	10	0.76 [0.67, 0.83]		0.91 [0.82, 0.96]	
Full vaccination rate			0.5943		0.0300
0%	39	0.70 [0.65, 0.74]		0.91 [0.88, 0.94]	
> 0%	3	0.74 [0.56, 0.87]		0.68 [0.35, 0.89]	
Guide			0.8268		0.7235
RSNA	22	0.71 [0.64, 0.77]		0.91 [0.85, 0.95]	
CO-RADS	20	0.69 [0.63, 0.76]		0.90 [0.83, 0.94]	

RSNA, Radiological Society of North America; CO-RADS, COVID-19 Reporting and Data System

^†^Likelihood ratio test between the bivariate models without and with a study-level characteristic

^‡^For continuous covariates, change in logit (sensitivity or specificity) per 1 unit increase are presented

**Fig. 3:**
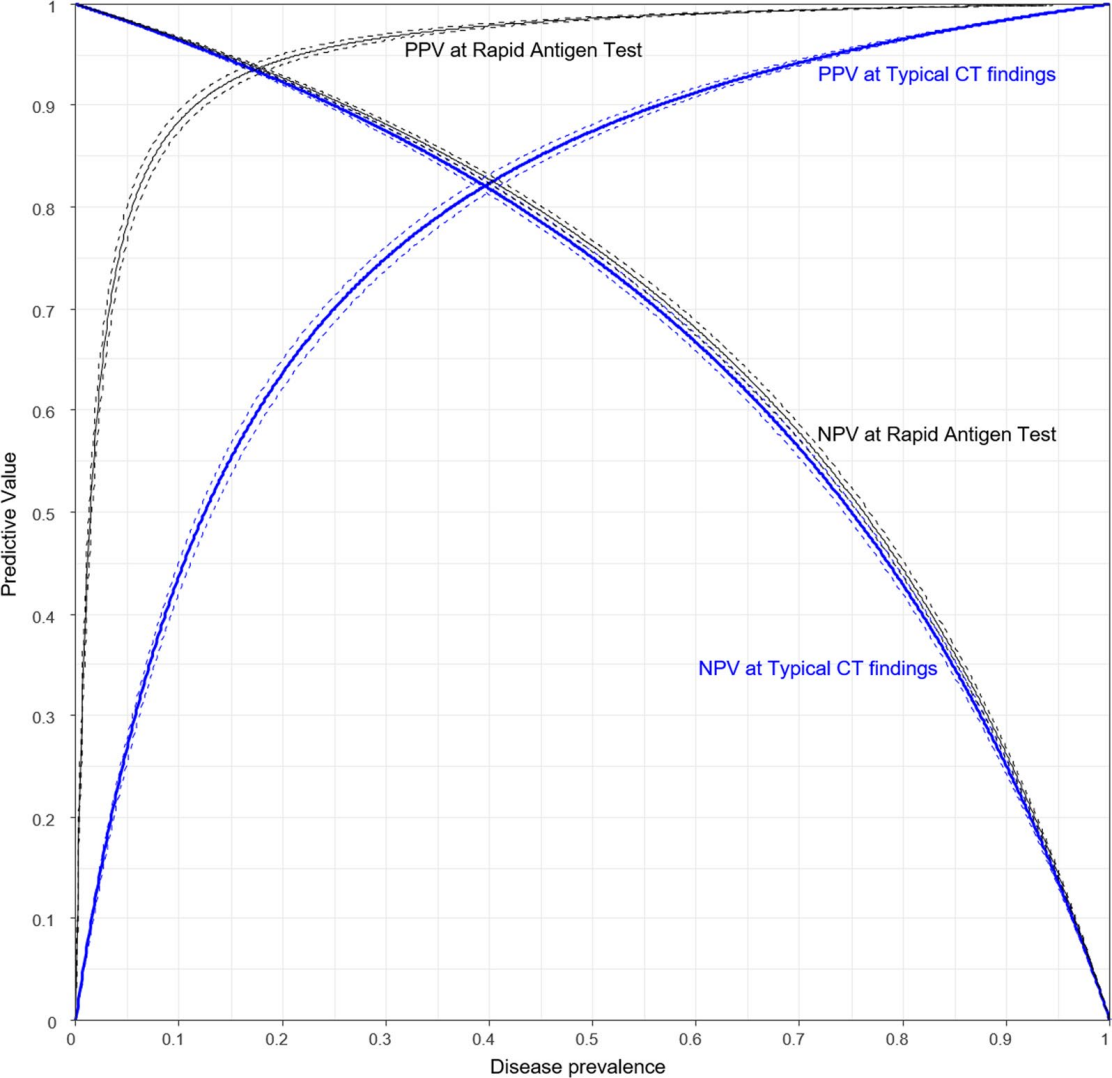
Estimated predictive values of typical CT findings and rapid antigen test (RAT). The black lines indicate predictive values and 95% confidence intervals for RAT, and the blue lines indicate predictive values and 95% confidence intervals for typical CT findings. Square indicates summary points of sensitivity and specificity, and solid black curvilinear line passing through summary point is the estimated summary ROC curve. Black dashed line and blue dashed line represent 95% confidence region and prediction region respectively. *Proportion of prediction region to area under ROC curve = 35.8%

A comparative analysis of diagnostic performance between typical CT findings and RAT is presented in Fig. 3. The PPV was significantly higher for RAT than for the typical CT findings, while there was no significant difference in NPV between RAT and the typical CT findings.

### Sensitivity analysis for the diagnostic performance of the typical CT findings of COVID-19

The results of a sensitivity analysis for 28 studies, including more than 50 subjects among both COVID-19 cases and non-COVID-19 cases, each, were similar to those of the primary analysis [9, 20–24, 26–28, 30, 33–38, 41, 45–48, 50–52, 54–57] (Additional file 1: Fig. 4). The pooled sensitivity was 70% (95% CI 65%, 75%; *I*^2^ = 94%) and the pooled specificity was 91% (95% CI 88%, 94%; *I*^2^ = 95%) for the typical CT findings.

### Interobserver agreement

Interobserver agreement for the typical CT findings was reported in 11 studies: seven studies reported diagnostic accuracy and the interobserver agreement together [9, 20, 22, 28, 37, 45, 51] and four studies solely reported the interobserver agreement [59, 60, 63, 64]. The reported κ values ranged from 0.52 to 0.93. The overall pooled estimate of κ values was 0.72 (95% CI 0.63, 0.81; *I*^2^ = 99%). However, the pooled estimate of κ values for the typical CT findings of the RSNA classification system (0.79; 95% CI 0.67, 0.91) was significantly higher than that of the CO-RADS system (0.64; 95% CI 0.55, 0.73) (*p* 0.05).

For the overall CT categories, the interobserver agreement was reported in 18 studies: 12 studies reported diagnostic accuracy and the interobserver agreement together [9, 20, 22, 25, 28, 36–39, 45, 51, 54] and six studies solely reported the interobserver agreement [59, 61–65]. They reported κ values ranged from 0.43 to 0.90. The pooled estimate of *κ* values was 0.67 (95% CI 0.61, 0.74; *I*^2^ = 99%). However, the pooled estimate of κ values for the overall CT findings of the RSNA classification system (0.74; 95% CI 0.65, 0.83) was significantly higher than that of the CO-RADS system (0.59; 95% CI 0.51, 0.67; *P* = 0.02) (Additional file 1: Fig. 5).

### Publication bias

There was no obvious publication bias in the studies reporting diagnostic accuracy of the typical CT findings of COVID-19 (Additional file 1: Fig. 6A). For interobserver agreement, there was a tendency for a lower degree of agreement to be shown as the precision of the κ value in the binomial classification decreased. There was no significant asymmetry in the overall CT classifications (Additional file 1: Fig. 6B).

## Discussion

This meta-analysis, which included 42 diagnostic performance studies from 18 developing and 24 developed countries, demonstrated that the pooled sensitivity was 70% (95% CI 65%, 74%) and the pooled specificity was 90% (95% CI 86%, 93%) for typical CT findings of COVID-19. In the meta-analysis of the RSNA classification system and CO-RADS in January 2021, the corresponding pooled estimates were 65% and 94% in the RSNA system (typical appearance; four studies from three countries) and 70% and 93% in CO-RADS (grade 5; six studies from three countries). We included a larger number of studies and merged the results of the typical findings in both systems. The diagnostic measure estimates of each system in the early observations were maintained in our results, while the 95% CIs became narrower. The pooled diagnostic performance did not differ by national income and region. Furthermore, the inter-reader agreement for dichotomizing CT findings into typical or not was substantial and higher than the categorical interpretation in both systems. These findings highlight that once typical findings of COVID-19 are defined in standardized reporting systems, radiologists can reproduce CT performance globally, despite geographic and resource variation.

In the early pandemic, the Fleischner Society advised using chest imaging for triaging suspects at risk of having moderate to severe COVID-19 when a point-of-care test was unavailable and resources were constrained [2]. However, whether chest CT could provide comparable accuracy for COVID-19 to point-of-care testing, as represented by RAT, remains underexplored. We compared the PPV and NPV of the typical CT findings and RAT based on pooled estimates for various disease prevalence rates: the NPV was almost identical between CT and RAT, and typical CT findings provided a lower PPV than RAT by up to 30–40%. When recollecting data from the massive Chinese surge of COVID-19 in early 2020, RAT was unavailable and PCR suffered from supply shortages and false-negative results, similar to shortages of large-scale laboratory testing capacities in low- and middle-income countries [66, 67]. The Chinese national guideline (trial version 5) temporarily used typical CT findings of viral pneumonia for making a clinical diagnosis of suspected COVID-19 cases in Wuhan and Hubei [68]. Given the pooled diagnostic CT performance, we may imagine why the first-line diagnosis/triage use of CT could be inevitably considered a practical supplemental option, although CT delivered radiation exposure [69] and it was unknown how well CT interpretation for typical COVID-19 findings was standardized in Wuhan and Hubei.

Chest CT findings can substantially overlap between COVID-19, influenza, and organizing pneumonia [70], and our findings on the pooled CT performance should be cautiously interpreted. Radiologists’ diagnostic CT accuracy for COVID-19 is low (~ 70%) if the three diseases have similar prevalence [70]. Fortunately, the incidence of influenza was historically low when COVID-19 predominated [71], and organizing pneumonia is an uncommon disease. Furthermore, vaccination [72] and the Omicron variant [73–75] can decrease diagnostic performance based on typical CT findings. Accordingly, the current pooled estimates are applicable to the early phase of the COVID-19 pandemic when vaccination was not sufficiently available and before the Omicron variant occurred.

The diagnostic accuracy of the typical findings among CT classification systems was almost identical between the RSNA and CO-RADS systems (*p* 0.72), in concordance with previous studies [10, 46, 59, 62]. In addition, higher CT categories showed higher diagnostic accuracy, similar to previous studies [10]. While the RSNA and CO-RADS systems have slightly different definitions of the typical category (such as the presence of subpleural sparing or thickened vessels), our study has demonstrated no significant difference in diagnostic accuracy between the two systems. Therefore, it is reasonable to consider the combined diagnostic ability of both systems in identifying typical COVID-19 pneumonia using a standardized classification approach. Meanwhile, the interobserver agreement was higher in the RSNA classification system than in the CO-RADS system (*κ* values, 0.79 vs 0.64). A previous study [46] surveyed preferences for the CT classification system and found that most participants preferred the RSNA system over the CO-RADS system. This is presumably because the RSNA classification system is more straightforward and user-friendly.

Our study had limitations. First, studies from low-income countries fundamentally lacking CT resources were not included in this study. Since few related studies are being conducted in low-income countries, further verification will be required based on the results of this study. Second, most of the studies were retrospectively designed and had a low vaccination rate, making it difficult to estimate the impact of vaccination on the diagnostic performance of the typical CT findings. Third, most studies did not provide information on the symptom presence, disease severity, and symptom onset of the included patients, although those factors could affect the diagnostic performance of CT findings.

In conclusion, the typical chest CT findings of COVID-19 based on standardized CT classification showed moderate sensitivity and high specificity globally, regardless of region and national income, and presented substantial interobserver agreement. If another pandemic occurs, radiology societies should prioritize providing standardized image interpretation for the pandemic disease’s typical findings as soon as possible. A standardized interpretation will play a crucial role in prompt diagnoses and triage until reference or point-of-care testing is sufficiently established.

## Supplementary Information

**Additional file 1**. Supplemental Material.

## Data Availability

Archived datasets that are publicly available were not included in our study.

## Abbreviations

CI: Confidence interval
CO-RADS: COVID-19 reporting and data system
QUADAS-2: Quality Assessment of Diagnostic Accuracy Studies 2
RAT: Rapid antigen test
ROC: Receiver operating characteristic
RSNA: Radiological Society of North America
RT-PCR: Reverse transcription polymerase chain reaction
