# Supplementary material for: Diagnostic performance of standardized typical CT findings for COVID-19: a systematic review and meta-analysis
Source: Insights Imaging. 2023 May 24;14:96. doi: 10.1186/s13244-023-01429-2 (PMC10206568; doi:10.1186/s13244-023-01429-2)

Diagnostic performance of standardized typical CT findings for COVID-19: A systematic review and meta-analysis

Study Data Extraction

The extracted study data were as follows: 1) patient characteristics (number of patients, mean age, sex distribution, inclusion period, and inclusion criteria); 2) study characteristics (authors, journal, affiliations, the region of the study, national income, disease prevalence [i.e., the proportion of the patients diagnosed with COVID-19 among those tested], and full vaccination rate); 3) the results of each diagnostic test (true-positive, false-positive, true-negative, and false-negative) regarding the results of RT-PCR assays; and 4) the interobserver agreement using the RSNA classification and the CO-RADS system.

For the reference standard of chest CT, any positive results and all negative results from initial or repeated RT-PCR assays were considered disease-positive and disease-negative findings, respectively. Any positive results from the repeated RT-PCR assays were regarded as disease-positive findings. Any discrepancies between two reviewers were resolved by consensus.

Statistical Analysis

Heterogeneity was assessed by a visual inspection of coupled forest plots and a scatter plot in the ROC plane, and by estimating the area of the prediction ellipse. Inconsistency was assumed if the individual studies substantially deviated from the summary ROC curve and the area of the prediction ellipse was large [1].

---

1. Reference: Macaskill PGC, Deeks J, Harbord R, Takwoingi Y (2010) Analysing and presenting results. In: Deeks JJ, Bossuyt PM, Gatsonis C, eds. Cochrane Handbook for Systematic Reviews of Diagnostic Test Accuracy. Version 10. The Cochrane Collaboration.

Supplementary Table 1. Study characteristics reporting interobserver agreement of standardized CT classification.

First author	Country	Continent	National income	Study design	Patient number	Inclusion period	Mean Age (y)	Male (%)	CT classification system	Number of reviewers	κ values for typical CT findings	κ values for overall CT categories
Abdel-Tawab	Egypt	Africa	Low-middle	Retrospective	359	Apr to May, 2020	45±17	50	RSNA	3	0.84	0.78
Atta	Egypt	Africa	Low-middle	Retrospective	991	Feb to July, 2020	45±16	56	CORADS	3	NS	0.78
Bellini	Italy	Europe	High	Retrospective	572	Mar to May, 2020	63±20	58	CORADS	12	0.60	0.43
Borges	Brazil	America	Upper-middle	Retrospective	175	Mar, 2020	43±21	59	RSNA	2	0.93	0.88
De Jaegere.	Netherlands	Europe	High	Retrospective	96	Mar, 2020	70	64	RSNA	2	NS	0.66
Dilek	Turkey	Europe	Upper-middle	Retrospective	178	Mar, 2020	47±16	57	CORADS	4	NS	0.73
Fujioka	Japan	Asia	High	Retrospective	154	Apr to June, 2020	61±19	66	CORADS	4	0.52	0.59
Inui	Japan	Asia	High	Retrospective	100	Jan to June. 2020	59±17	76	RSNA	8	NS	0.63
Kavak	Turkey	Europe	Upper-middle	Retrospective	903	July, 2020	49±17	49	RSNA	2	0.93	0.88
Kosar	Turkey	Europe	Upper-middle	Retrospective	209	Mar to May, 2020	57	55	CORADS	4	NS	0.57
Kuokawa	Japan	Asia	High	Retrospective	154	Apr to May, 2020	65	70	RSNA	6	NS	0.57
Nair	India	Asia	Low-middle	Retrospective	164	Apr to July, 2020	49±15	84	CORADS	6	0.79	0.68
Prokop	Netherlands	Europe	High	Retrospective	105	Mar, 2020	62±16	58	CORADS	8	0.68	0.47
Rocha	Brazil	America	Upper-middle	Retrospective	160	May to Jun, 2020	59	53	RSNA	4	0.65	0.53
Silva	Chile	America	High	Retrospective	240	Mar to Apr, 2020	48±156	37	RSNA	2	NS	0.90
Byrne	Canada	America	High	Retrospective	303	Mar, 2020	48	52	RSNA	3	0.81	NS
Fonseca	Brazil	America	Upper-middle	Retrospective	100	Mar to May, 2020	54±18	52	RSNA	3	NS	0.89
Hadied	USA	America	High	Retrospective	210	Mar to Apr, 2020	NS	NS	RSNA	5	0.57	0.67
Sushentsev	Russia	Europe	Upper-middle	Retrospective	1110	Mar to Apr, 2020	47 (median)	56	CORADS	3	0.58	0.55

Abbreviations: NS = not specified.

Supplementary Figure 1A. Typical CT imaging (CO-RADS 5) for COVID-19.

Non-enhanced chest CT axial images of the lungs in a 67-year-old woman with a positive RT-PCR (A-D) show bilateral, multifocal and peripheral consolidations with ground-glass opacities.

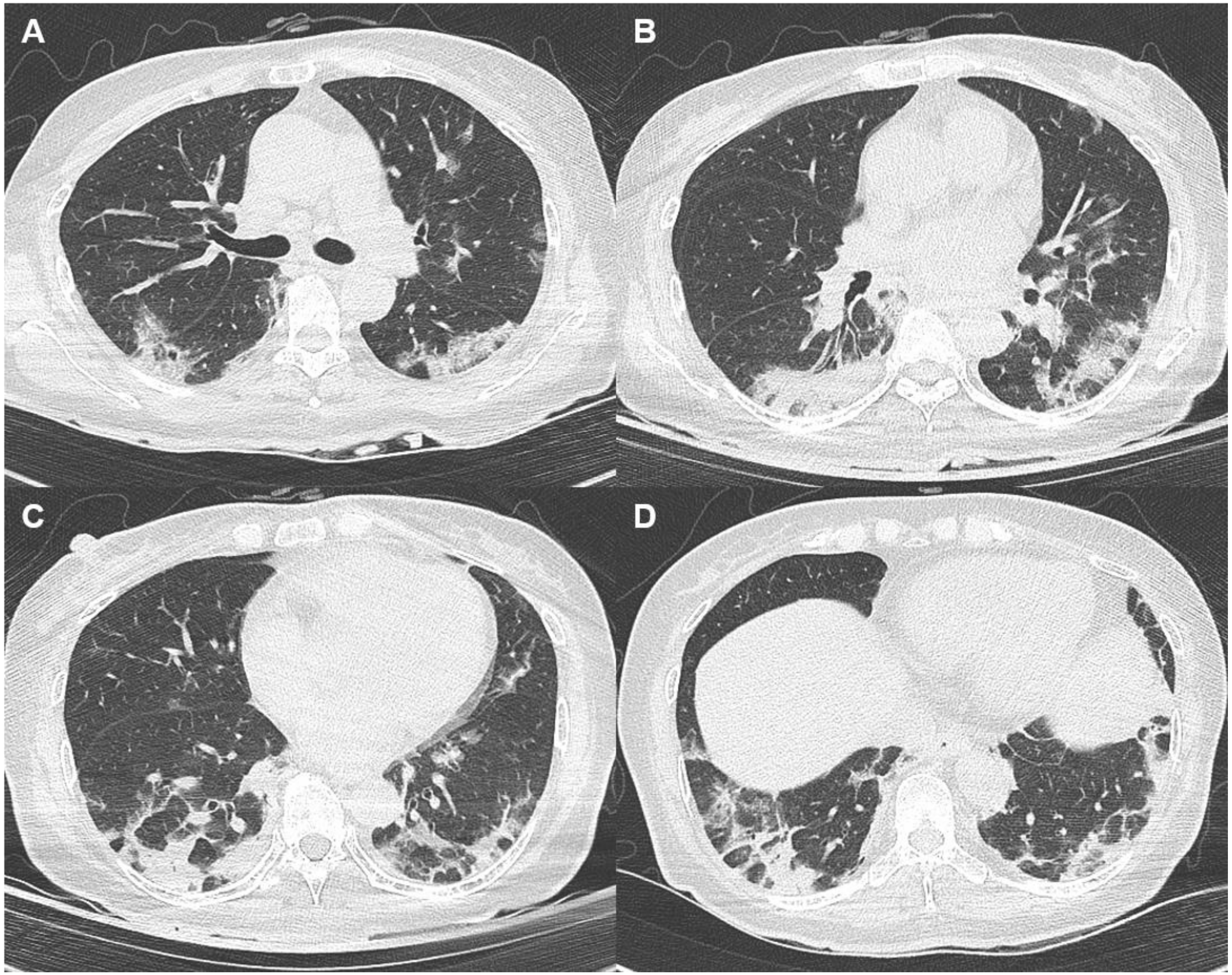

Supplementary Figure 1B. Indeterminate CT imaging (CO-RADS 3-4) for COVID-19.

Non-enhanced chest CT axial images of the lungs in a 49-year-old man show relatively unilateral ground-glass opacities with consolidation and no peripheral distribution.

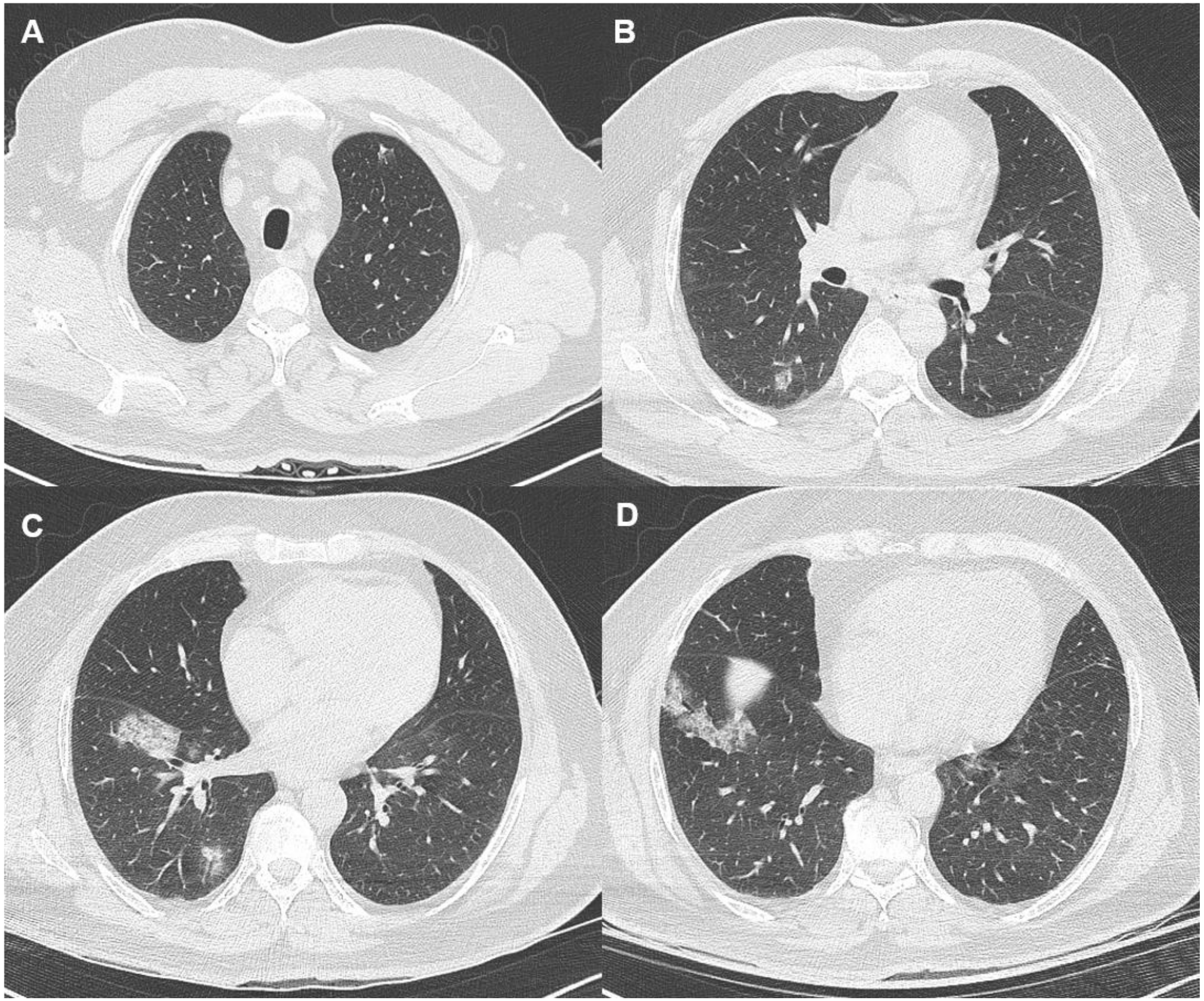

Supplementary Figure 1C. Atypical CT imaging (CO-RADS 2) for COVID-19.

Non-enhanced chest CT axial images of the lungs in an 83-year-old woman show centrilobular nodules with micronodules and segmental consolidations in both lungs.

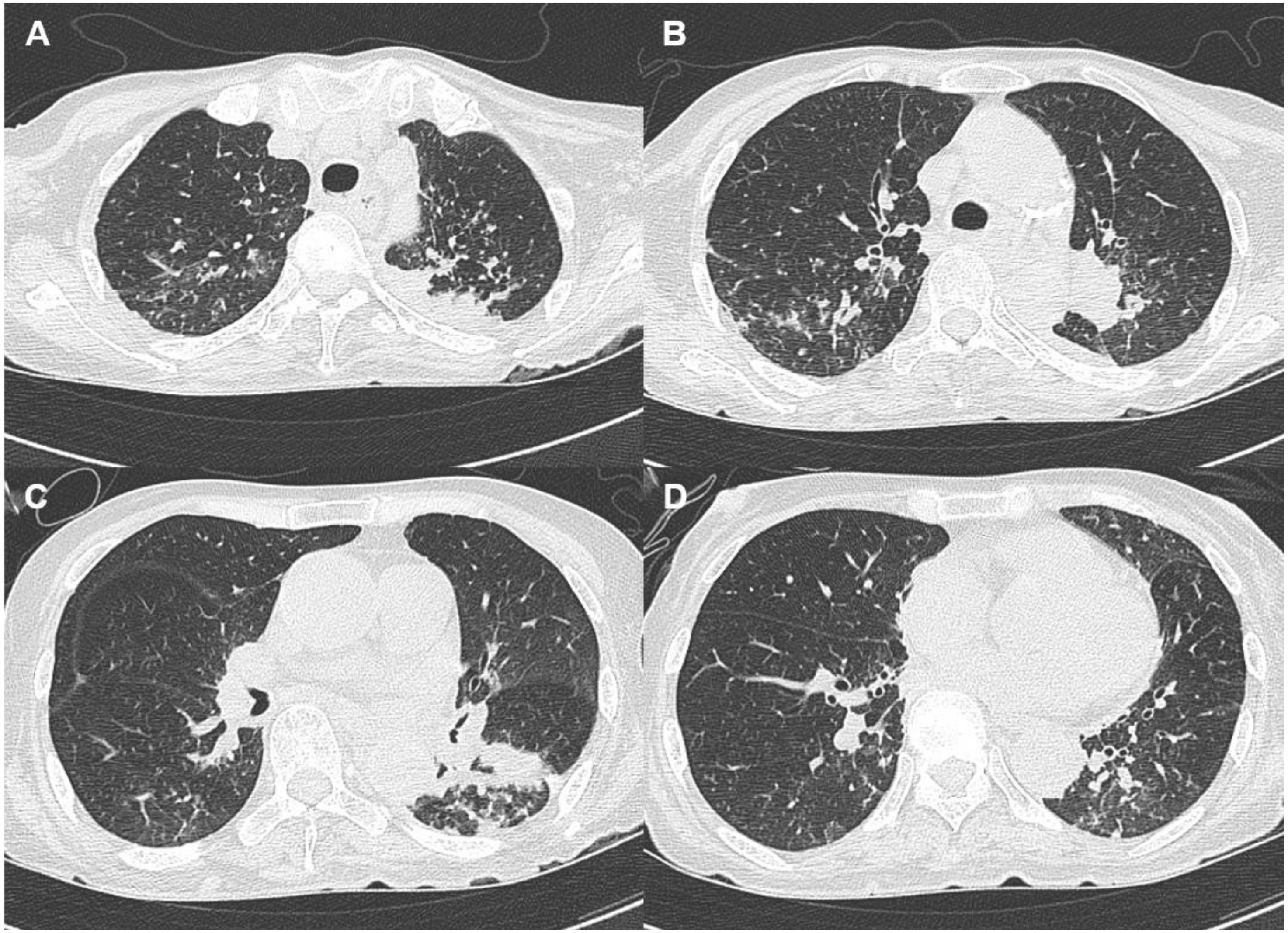

Supplementary Figure 2. Quality Assessment of Diagnostic Accuracy Studies 2 (QUADAS-2) quality assessments of included studies.

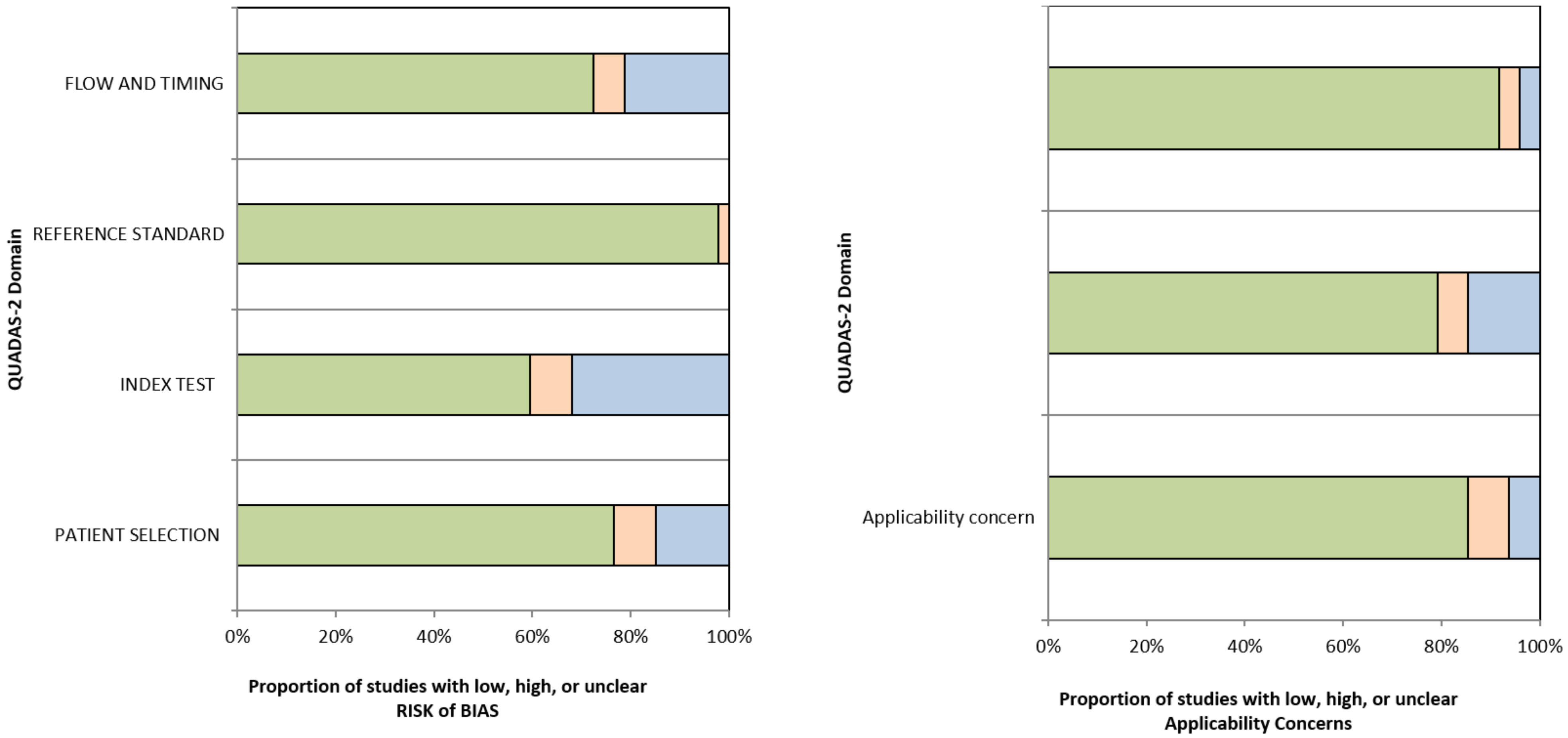

Supplementary Figure 3. Coupled forest plots for the diagnostic accuracy of typical CT findings in studies according to the prevalence of COVID-19.

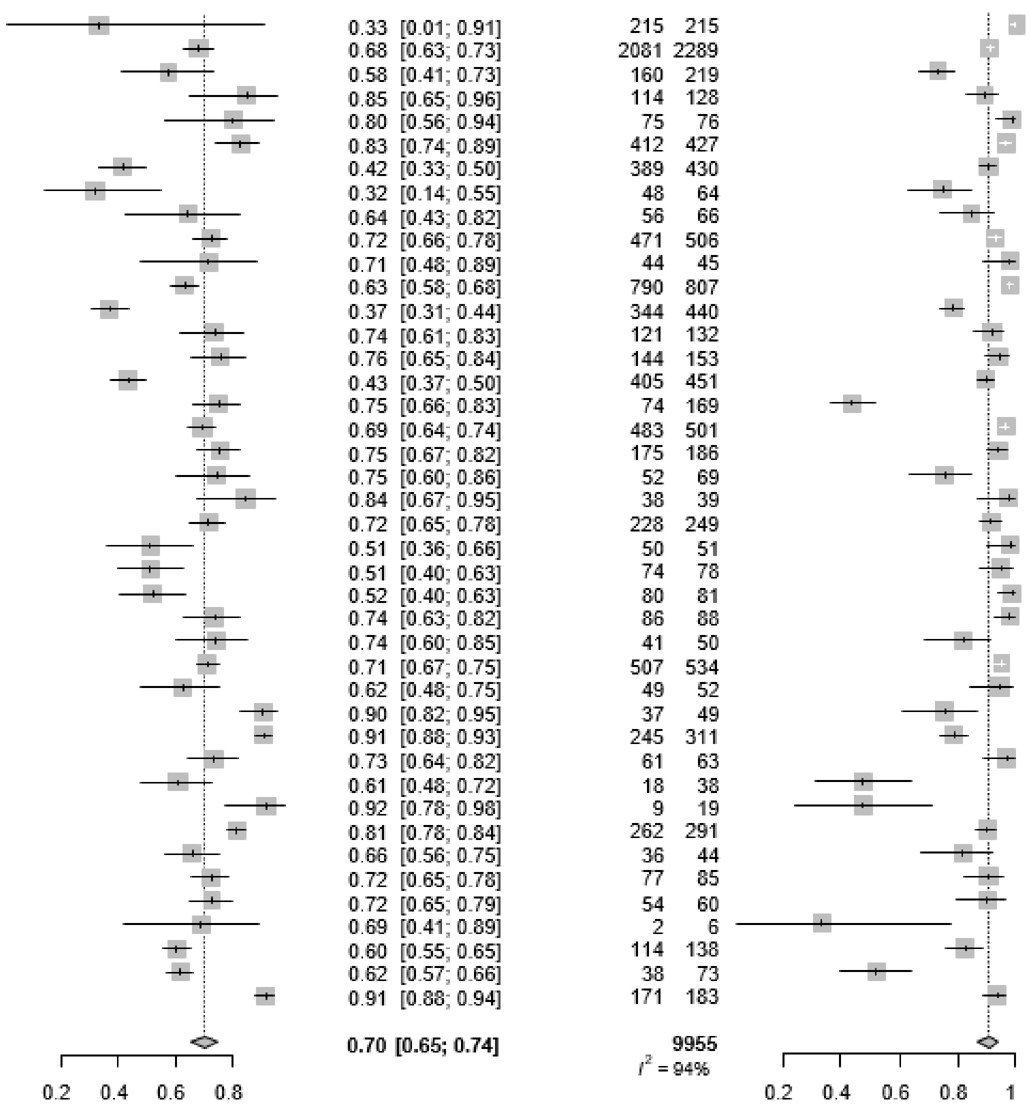

Study	Prevalence(%)	Events	Total	Sensitivity [95% CI]	Events	Total	Specificity [95% CI]
16 G¡¡m¡¡s et al. 2021 Turkey	1.40	1	3	0.33 [0.01; 0.91]	215	215	1.00 [0.98; 1.00]
17 Hammer et al. 2022 USA	13.00	224	329	0.68 [0.63; 0.73]	2081	2289	0.91 [0.90; 0.92]
26 Majeed et al. 2020 UK	15.40	23	40	0.58 [0.41; 0.73]	160	219	0.73 [0.67; 0.79]
23 Kuokawa et al. 2021 Japan	16.90	22	26	0.85 [0.65; 0.96]	114	128	0.89 [0.82; 0.94]
14 Gross et al. 2021 Germany	21.00	16	20	0.80 [0.56; 0.94]	75	76	0.99 [0.93; 1.00]
40 Valentin et al. 2022 Germany	21.20	90	109	0.83 [0.74; 0.89]	412	427	0.96 [0.94; 0.98]
5 Bellini et al. 2021 Italy	24.80	59	142	0.42 [0.33; 0.50]	389	430	0.90 [0.87; 0.93]
2 Abdolarhimzadeh et al. 2021 Iran	25.60	7	22	0.32 [0.14; 0.55]	48	64	0.75 [0.63; 0.85]
4 Barbosa et al. 2020 Brazil	27.50	16	25	0.64 [0.43; 0.82]	56	66	0.85 [0.74; 0.92]
24 Lieveld et al. 2021 Netherlands	31.80	170	235	0.72 [0.66; 0.78]	471	506	0.93 [0.91; 0.95]
37 Shirota et al. 2021 Japan	31.80	15	21	0.71 [0.48; 0.89]	44	45	0.98 [0.88; 1.00]
31 ¡¡zer et al. 2021 Turkey	32.00	239	379	0.63 [0.58; 0.68]	790	807	0.98 [0.97; 0.99]
19 Inanc et al. 2022 Turkey	33.80	83	225	0.37 [0.31; 0.44]	344	440	0.78 [0.74; 0.82]
6 Berkel et al. 2021 Belgium	34.00	50	68	0.74 [0.61; 0.83]	121	132	0.92 [0.86; 0.96]
38 Silva et al. 2021 Chile	36.30	66	87	0.76 [0.65; 0.84]	144	153	0.94 [0.89; 0.97]
41 Vicini et al. 2021 Italy	36.80	114	263	0.43 [0.37; 0.50]	405	451	0.90 [0.87; 0.92]
30 ¡¡zel etal. 2021 Turkey	39.60	83	111	0.75 [0.66; 0.83]	74	169	0.44 [0.36; 0.52]
11 De Smet at al. 2021 Belgium	41.70	248	358	0.69 [0.64; 0.74]	483	501	0.96 [0.94; 0.98]
18 Hermans et al. 2020 Netherlands	41.70	100	133	0.75 [0.67; 0.82]	175	186	0.94 [0.90; 0.97]
39 Turcato et al. 2021 Italy	42.50	38	51	0.75 [0.60; 0.86]	52	69	0.75 [0.64; 0.85]
27 Miranda et al. 2020 Brazil	45.10	27	32	0.84 [0.67; 0.95]	38	39	0.97 [0.87; 1.00]
9 Ciccarese et al. 2020 Italy	45.90	151	211	0.72 [0.65; 0.78]	228	249	0.92 [0.87; 0.95]
10 De Jaegere et al. 2020 Netherlands	47.00	23	45	0.51 [0.36; 0.66]	50	51	0.98 [0.90; 1.00]
13 Fujioka et al. 2020 Japan	49.40	39	76	0.51 [0.40; 0.63]	74	78	0.95 [0.87; 0.99]
35 Rocha et al. 2022 Brazil	49.40	41	79	0.52 [0.40; 0.63]	80	81	0.99 [0.93; 1.00]
7 Borges Da Silva Teles et al. 2021 Brazil	49.70	64	87	0.74 [0.63; 0.82]	86	88	0.98 [0.92; 1.00]
20 Inui et al. 2022 Japan	50.00	37	50	0.74 [0.60; 0.85]	41	50	0.82 [0.69; 0.91]
36 Schalekamp et al. 2021 Netherlands	50.00	381	536	0.71 [0.67; 0.75]	507	534	0.95 [0.93; 0.97]
34 Prokop et al. 2020 Netherlands	50.50	33	53	0.62 [0.48; 0.75]	49	52	0.94 [0.84; 0.99]
32 Palmisano et al. 2021 Italy	54.90	84	93	0.90 [0.82; 0.95]	37	49	0.76 [0.61; 0.87]
46 Falaschi et al. 2020 Italy	59.80	419	462	0.91 [0.88; 0.93]	245	311	0.79 [0.74; 0.83]
28 Nair et al. 2021 India	61.60	74	101	0.73 [0.64; 0.82]	61	63	0.97 [0.89; 1.00]
49 Guner et al. 2021 Turkey	63.50	40	66	0.61 [0.48; 0.72]	18	38	0.47 [0.31; 0.64]
53 Martinez-Fierro et al. 2022 Mexico	65.50	33	36	0.92 [0.78; 0.98]	9	19	0.47 [0.24; 0.71]
21 Kavak et al. 2021 Turkey	67.80	497	612	0.81 [0.78; 0.84]	262	291	0.90 [0.86; 0.93]
42 Yassin et al. 2022 Egypt	69.40	66	100	0.66 [0.56; 0.75]	36	44	0.82 [0.67; 0.92]
29 O¡¯ Neill et al. 2021 Canada	69.50	140	194	0.72 [0.65; 0.78]	77	85	0.91 [0.82; 0.96]
22 Ko¡¡ar Tun¡¡ et al. 2021 Turkey	71.30	108	149	0.72 [0.65; 0.79]	54	60	0.90 [0.79; 0.96]
51 Lang et al. 2020 USA	73.00	11	16	0.69 [0.41; 0.89]	2	6	0.33 [0.04; 0.78]
8 Cengel et al. 2021 Turkey	74.20	239	396	0.60 [0.55; 0.65]	114	138	0.83 [0.75; 0.89]
33 Palwa et al. 2021 Pakistan	85.40	263	427	0.62 [0.57; 0.66]	38	73	0.52 [0.40; 0.64]
15 Guimaraes et al. 2022 Brazil	89.00	282	309	0.91 [0.88; 0.94]	171	183	0.93 [0.89; 0.97]
**Random effects model**			**6777**	**0.70 [0.65; 0.74]**		**9955**	**0.90 [0.86; 0.93]**
Heterogeneity: I^2 = 92%					I^2 = 94%		

Supplementary Figure 4. Coupled forest plots for the diagnostic accuracy of typical CT findings in studies with more than 50 subjects in both COVID-19 cases and non-COVID-19 patients.

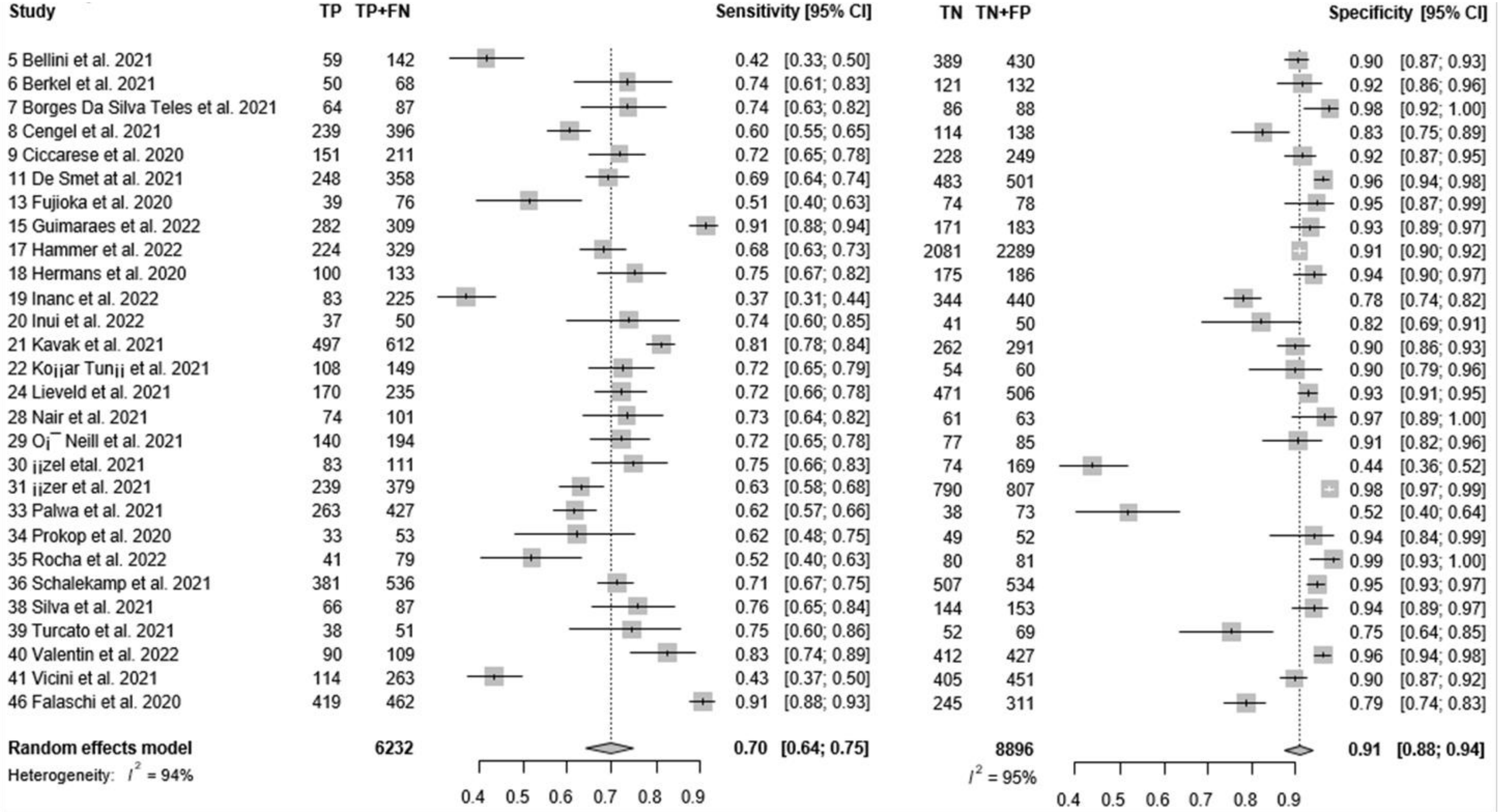

Supplementary Figure 5. Forest plots of interobserver agreement for typical findings and overall CT categories.

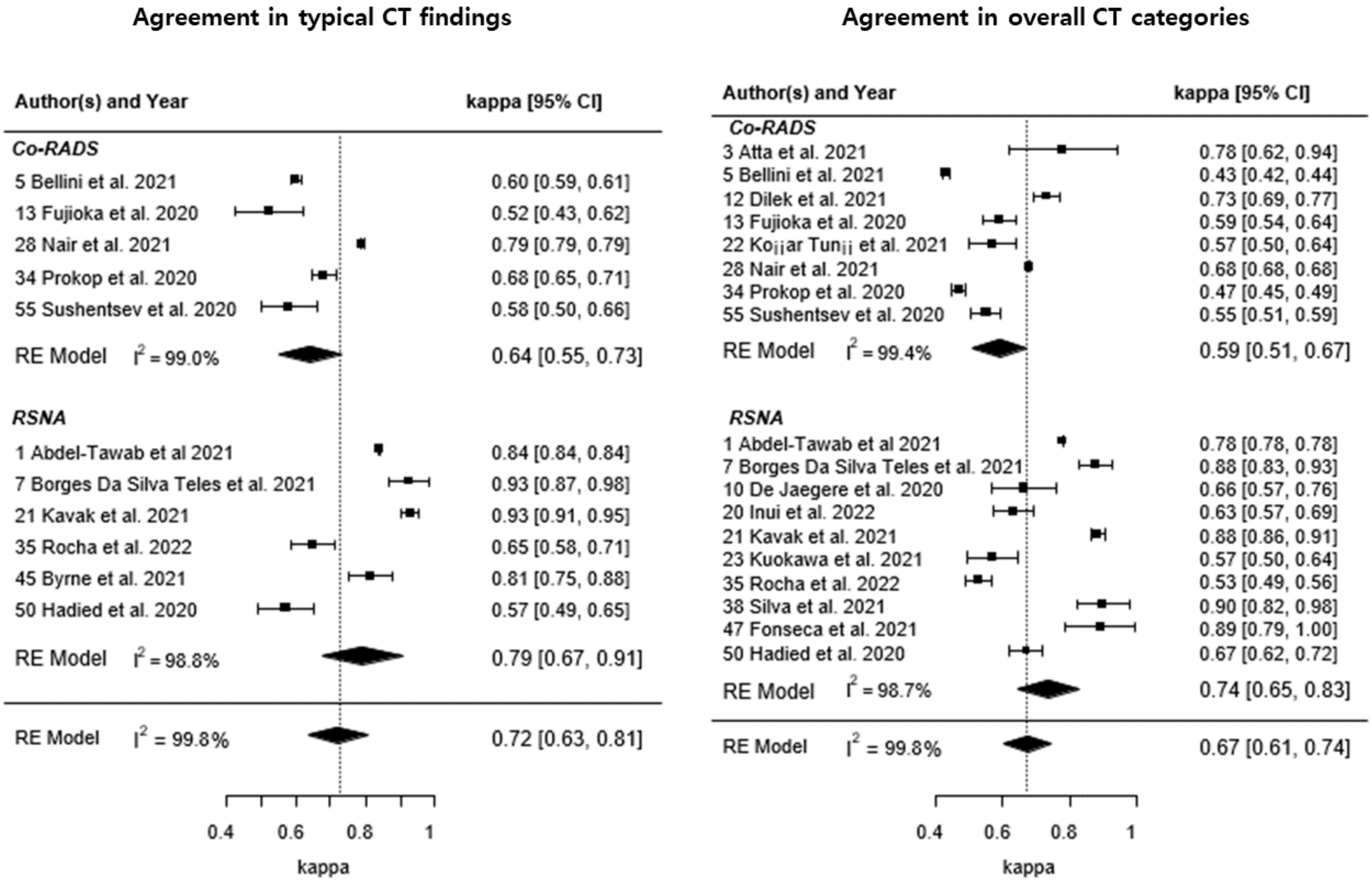

Supplementary Figure 6A. Deeks' regression test for diagnostic accuracy.

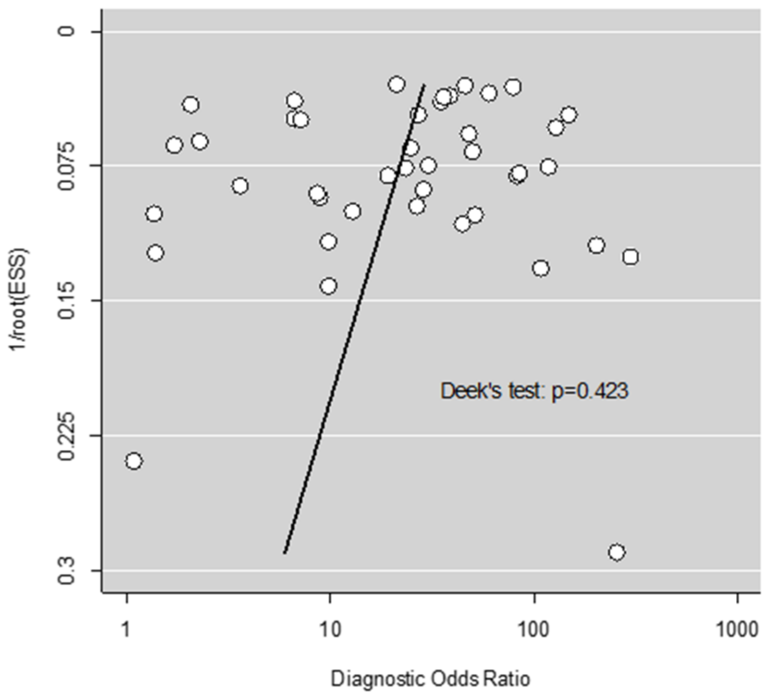

Supplementary Fig. 6B. Funnel plots for interobserver agreement.

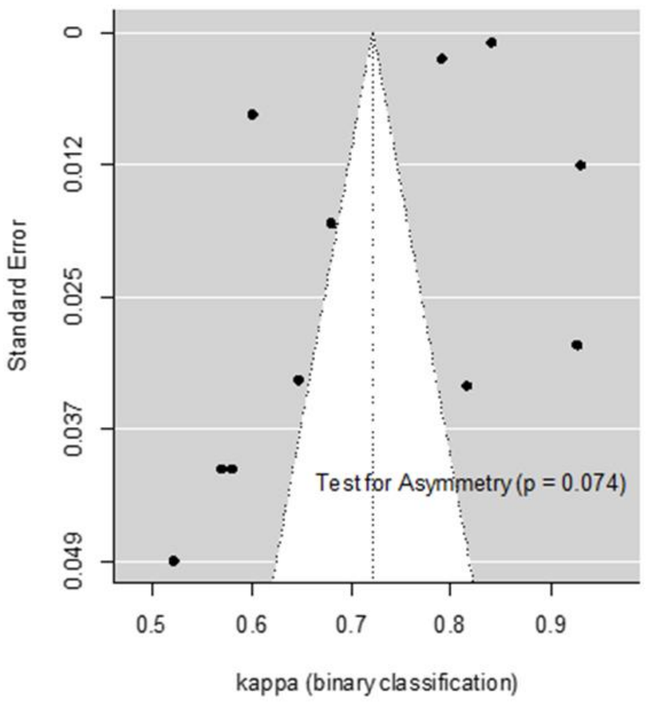

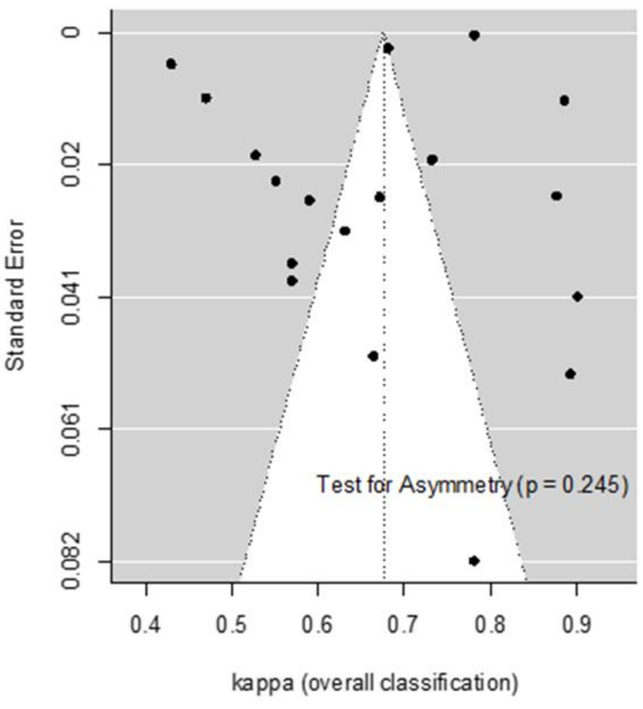

Supplement: Supplementary file 1 — Additional file 1. Supplemental Material. [file 13244_2023_1429_MOESM1_ESM.pdf]
